# Anxiety and Depression in a Sample of Iranian Patients With Irritable Bowel Syndrome

**Published:** 2013

**Authors:** Neda Farzaneh, Mehdi Ghobakhlou, Bijan Moghimi-Dehkordi, Nosratollah Naderi, Farbod Fadai

**Affiliations:** 1 Department of Psychiatry, University of Social Welfare and Rehabilitation Science, Tehran, Iran.; 2 Research Center for Gastroenterology and Liver Disease, ShahidBeheshti University of Medical Sciences, Tehran, Iran.

**Keywords:** Anxiety, Depression, Irritable Bowel Syndrome (IBS), Psychiatric Symptoms, Rome III Criteria

## Abstract

**Objective: **Previous studies have shown that up to 50% to 70% of patients with irritable bowel syndrome (IBS) who seek treatment have psychiatric comorbidity. The aim of this study was to report the history of anxiety and depression in IBS patients.

**Methods: **The study, designed as a cross-sectional, was performed in a consecutive sample of individuals diagnosed with IBS in the Gastroenterology Clinic of Taleghani Hospital, Tehran province, Iran from October 2010 to October 2011. IBS was diagnosed according to the Rome III criteria**. **All participants were asked about perceived psychiatric symptoms such as depression and anxiety in the past six months.

**Results: **153 treatment-seeking IBS patients were entered to the study. The mean age of IBS patients with history of psychiatric symptoms (33.9 years) was relatively lower than those without this history (39.3 years); p< 0.05. There was a significant difference regarding mean age of diarrhea-predominant IBS (IBS-D) patients with history of psychiatric symptoms (26.8 years) and without such history (37.9 years); p< 0.05. However, such a difference was not seen among other types of IBS. The most frequent psychiatric symptoms were reported by the constipation-predominant IBS (IBS-C) patients followed by mixed-IBS (IBS-M) and the least rate was seen in IBS-D.

**Conclusion:** Approximately half of IBS patients reported anxiety and/or depression. IBS-C patients experienced higher proportions of anxiety and depression.

**Declaration of interest:** None.

## Introduction

The association between psychiatric disturbance and gastrointestinal (GI) disorders such as Irritable Bowel Syndrome (IBS) is well established (1,2). IBS, the most common disorder seen in gastroenterology practice (-), is a chronic condition of unknown etiology that affects approximately 6 to 18% of the Western population with a marked female preponderance (-). Patients with IBS are suffering from a variety of GI complaints, as well as, associated symptoms like headache, dysuria, fibromyalgia, anxiety, depression, and chronic fatigue syndrome(13). Anxiety, depression and somatoform disorders are the most prevalent psychiatric conditions linked to IBS. It is reported that at least one of these disorders is present in approximately 40 to 60 percent of IBS patients visited by physicians in gastroenterology clinics (14). Results from some studies with population-based design have reported lower rates of psychiatric symptoms in non-consulters IBS patients (i.e., individuals who do not consult with any physician) (15). While a number of IBS patients feel that their psychiatric disorders is the result of the intrusive nature of their IBS, others think that their psychiatric disorders are related to the development of their GI symptoms. However, approximately half of patients with a psychiatric disorder develop the condition before the onset of GI symptoms, and psychiatric symptoms start at the same time in most of the remaining fifty percent (16,17).

Although it is unlikely that psychiatric disorders are the single cause of IBS, they may be important cofactors in the development of the illness in susceptible people. A number of studies support this hypothesis, having found that the presence of upper GI symptoms in IBS patients increases the incidence and severity of psychopathology (18,19).Gwee at al. (20)found that those who subsequently developed IBS had higher levels of anxiety, depression, somatization and neurotic personality traits than those who did not develop the illness.

Though no study has evaluated yet the possible relationship between IBS and psychiatric symptoms in developing countries like Iran, this study aimed to investigate the co-occurrence of IBS and perceived psychiatric symptoms such as anxiety and depression. This study is a preliminary effort to examine the nature of this relationship by using the cross-sectional design.

## Materials and Methods

The study, designed as a cross-sectional, was performed in a consecutive sample of individuals diagnosed with IBS in the Gastroenterology Clinic of Taleghani Hospital, Tehran province, Iran from October 2010 to October 2011. 

IBS was diagnosed according to the Rome III questionnaire that have been previously translated to Persian and validated in a random sample of Iranian population (12,-).

IBS was defined as presence of abdominal pain or discomfort for at least 2 days a week for the previous 3 months, accompanied by at least 2 of the following features: (a) it improved with defecation, (b) it was associated with a change in the frequency of bowel movements, or (c) it was associated with a change in the appearance of the stool. The onset of symptoms had to be at least 6 months prior to the enrollment. The patients who were diagnosed as having IBS were further categorized into constipation-predominant IBS (IBS-C) if they had hard or lumpy stools with no loose, watery mushy or watery stools in the past 3 months; diarrhea-predominant IBS (IBS-D) if they had loose, mushy or water stools in the last 3 months with no hard or lumpy stools and mixed-IBS (IBS-M) if they had both loose and hard stools in the past 3 months. 

All participants were asked about perceived psychiatric symptoms such as depression and anxiety in the past six months. The patients were also questioned about their age and gender. The protocol was considered and approved by the Ethics Committee and informed written consent was obtained from every subject.

Continuous variables were expressed as mean ±SD and compared by use of the Student's t test and one-way ANOVA (analysis of variance), whereas discrete variables were given as absolute values and percentages. The χ2 test was used to compare proportions. All statistical analyses were carried out using the SPSS version 13.0 software (SPSS, Chicago, IL, USA). Differences were considered statistically significant if the P-value was less than 0.05.

## Results

A total of 153 treatment-seeking individuals visited by a gastroenterologist and diagnosed by IBS were entered to the study. There were 95 females (62.1%). The majority of participants aged between 30 to 45 years (n= 63, 41.2%) followed by ages lower that 30 (n= 56, 36.6%) and higher than 45 years (n= 34, 22.2%). 

Mean±SD age of the patients was 36.3±13.5 years. Mean±SD age of IBS patients with history of psychiatric symptoms (33.9±11.8 years) was relatively lower than those without such history (39.3±14.9 years) (p=0.016). There was a statistically significant difference regarding mean ±SD age in IBS-D patients with and without past history of psychiatric symptoms (26.8±3.8 and 37.9±13.1, respectively; p=0.01). But such a difference was not seen among other types of IBS.

Table 1 shows the frequency of the patients' reports on psychiatric symptoms in different subtypes of IBS. The most frequent psychiatric symptoms were reported by the IBS-C patients (64.3%) followed by IBS-M and IBS-D. There was a significant difference among types of IBS regarding presence or absence of psychiatric symptoms (p< 0.05).It seems that patients with history of anxiety or depression symptoms tend to be IBS-C.

Approximately 64% of males and 50% of females under study reported history of psychiatric symptoms (p>0.05). Table2 delineates the detailed frequency of psychiatric symptoms history between different subtypes of IBS according to age and gender.

## Discussion

In this clinic-based study, we investigated cross-sectional relationship between perceived depression and anxiety symptoms with IBS. 

We found that individuals with history of psychiatric symptoms tend to suffer from in onstipation-dominant IBS. Approximately two third of males and half of females reported history of psychiatric symptoms in the last six-month, but this observed difference was not statistically significant.

Though the study revealed interesting facts about relationship between these two common psychiatric symptoms (perceived depression and anxiety) and IBS in Iran, the results of this study must be interpreted with caution because, first, this research was performed in a hospital gastroenterology clinic and therefore the study population may not be a representative sample of Iranian IBS patients. Second, evaluation of psychological disorders was not on the basis of standard questionnaires and rather was based on subjective reports and therefore this may cause a serious bias in the results (i.e., recall bias). 

The prevalence of perceived psychiatric symptoms in the present study was approximately 64% in men and 50% in women. Our results on prevalence of perceived psychiatric symptoms in women are keeping with one Iranian study which reported that 35.6% of men and 49.6% of women were depressed (24). High rates of depressive symptoms were reported as 55% of nursing students in Thailand (25). Our findings are in contrast with one previous study showing a greater prevalence of perceived depression in women compared to men (26). 

In line with other studies, we found evidence for a link between perceived psychiatric symptoms andIBS. An association between IBS and psychiatric illness has been observed in 27% to 60% of patients diagnosed as having a comorbid psychiatric disorder (-). Although it is mentioned that no particular psychiatric disorder has been correlated with IBS, but depression, anxiety, and somatoform disorders have been the most frequently diagnosed disorders in those patients (32) and approximately 40% to 60% of IBS patients visited at hospital-based gastroenterology clinics reported one or more of the mentioned disorders (14, 33). Co-occurrence of IBS with psychiatric disorders have been reported about 29% (34) for major depression, 60% for dysthymia (35), 19% for schizophrenia (36), and 44% for panic disorder (37). Whitehead and Crowell (38) with combination data from four studies estimated that the lifetime prevalence of depression and anxiety in IBS patients are approximately 20% and 15%, respectively. Miller et al. (39) revealed that somatization disorder, is also common, being present in up to 40% of IBS patients seen in hospital outpatient clinics. Tirkas et al. (40)pointed out that anxiety and depression, as well as somatoform disorders, appear to be highly correlated with symptom severity and poor response to treatment in IBS patients.

**Table 1:** Frequency (percentage) of anxiety and depression in different subtypes of IBS

	**History of anxiety and/or depression**		**P-value df χ** ^2^
**IBS types**	**No**	**Yes**	
**IBS-Constipation (N= 80)**	26 (37.7%)	54 (64.3%)	0.001 1 10.75
**IBS-Diarrhea (N= 22)**	13 (18.8%)	9 (10.7%)	0.15 1 2.02
**IBS-Mixed (N= 51)**	30 (43.5%)	21 (25.0%)	0.01 1 5.82
**Total **	69 (100.0%)	84 (100.0%)	

**Table 2:** Frequency of positive or negative history of psychiatric symptoms between different subtypes of IBS according to age and gender

	**History of Psychiatric symptoms**								
	**No **			**Yes **			**Total**		
	**IBS-C**	**IBS-D**	**IBS-M**	**IBS-C**	**IBS-D**	**IBS-M**	**IBS-C**	**IBS-D**	**IBS-M**
**Age (years)** **(Mean±SD)**	39.4±13.8	37.9±13.1^*^	39.7±19.8	34.4±12.0	26.8±3.8^*^	36.0±12.9	36.0±12.8	33.4±11.6	38.2±15.3
**<30**	7(31.8%)	4(18.2%)	11(50.0%)	20(58.8%)	8(23.5%)	6(17.6%)	27(48.2%)	12(21.4%)	17(30.4%)
**30-45**	10(37.0%)	6(22.2%)	11(40.7%)	25(69.4%)	1(2.8%)	10(27.8%)	35(55.6%)	7(11.1%)	21(33.3%)
**>45**	9(45.0%)	3(15.0%)	8(40.0%)	9(64.0%)	0(0%)	5(35.7%)	18(52.9%)	3(8.8%)	13(38.2%)
**Gender**									
**Male **	7(33.3%)	4(19.0%)	10(47.6%)	23(62.2%)	4(10.8%)	10(27.0%)	30(51.7%)	8(13.8%)	20(34.5%)
**Female **	19(36.9%)	9(18.8%)	20(41.7%)	31(66.0%)	5(10.6%)	11(23.4%)	50(52.6%)	14(14.7%)	31(32.6%)
**Total**	26(37.7%)	13(18.8%)	30(43.5%)	54(64.3%)	9(10.7%)	21(25%)	80(52.3%)	22(14.4%)	51(33.3%)

Statistical difference among mean age of IBS-D patients with or without history of psychiatric symptoms (p<0.05)

We found that the most frequent psychiatric symptoms were reported by the IBS-C patients followed by IBS-M and minimized to IBS-D (P< 0.05). It seems that patients with a history of anxiety or depression symptoms tend to be IBS-C. Cann et al. (41) revealed that under laboratory conditions stress has shown a capability to induce changes in bowel motility , so that transit may be delayed in IBS-C or quickened by stress in IBS-D. It is suggested that stress involves a complex interaction of the enteric nervous system, the hypothalamic-pituitary-adrenal axis, and the gut neuroinflammatory cells; this includes the mast cells especially, which are innervated directly via connections from the central nervous system (42,43).  In line with our results, Eriksson et al. and Aggarwal  et al. have found that IBS-C patients were more psychologically distressed, with higher degrees of depression and anxiety but IBS-D patients experienced less psychological symptoms (13,44). 

In conclusion, approximately half of IBS patients reported anxiety and/or depression. IBS-C patients experienced higher proportion of psychological disorders compared to other categories of IBS. IBS-D patients with history of psychiatric symptoms have been reached the disease in early ages. These data suggest that screening, detection and treatment of psychiatric illness among IBS patients by the gastroenterologist is important. Future studies using established instruments for accurate evaluation and diagnosis of psychiatric disturbances is suggested.

***Limitations of the study***

In addition to selection bias and lack of a standard questionnaire which have been mentioned earlier, the most important limitation of this study is when we asked the patients for drug history, the majority of participants did not reported any exact information about past drug history Constipation may be due to anticholinergic properties of psychotropic medications. 

## Authors' Contributions

 NF conceived and designed the evaluation and helped to draft the manuscript. MGh participated in designing the evaluation and patient finding. BMD performed the statistical analysis and revised the manuscript. NN advised for gathering the clinical data and interpreted them and revised the manuscript. FF supervised the study. All authors read and approved the final manuscript.

## References

[1] Folks DG (2004). The interface of psychiatry and irritable bowel syndrome. Curr Psychiatry Rep.

[2] Henningsen P, Herzog W (2008). Irritable bowel syndrome and somatoform disorders. J Psychosom Res.

[3] Ghannadi K, Emami R, Bashashati M, Tarrahi MJ, Attarian S (2005). Irritable bowel syndrome: an epidemiological study from the west of Iran. Indian J Gastroenterol.

[4] Xiong LS, Chen MH, Chen HX, Xu AG, Wang WA, Hu PJ (2004). A population-based epidemiologic study of irritable bowel syndrome in South China: stratified randomized study by cluster sampling. Aliment Pharmacol Ther.

[5] Jun DW, Park HY, Lee OY, Lee HL, Yoon BC, Choi HS (2006). A population-based study on bowel habits in a Korean community: prevalence of functional constipation and self-reported constipation. Dig Dis Sci.

[6] Safaee A, Moghimi-Dehkordi B, Pourhoseingholi MA, Vahedi M, Habibi M, Pourhoseingholi A (2011). Bloating in irritable bowel syndrome. Gastroenterol Hepatol Bed to Bench.

[7] Sorouri M, Pourhoseingholi MA, Vahedi M, Safaee A, Moghimi-Dehkordi B, Pourhoseingholi A (2010). Functional bowel disorders in Iranian population using Rome III criteria. Saudi J Gastroenterol.

[8] Ladep NG, Okeke EN, Samaila AA, Agaba EI, Ugoya SO, Puepet FH (2007). Irritable bowel syndrome among patients attending General Outpatients' clinics in Jos, Nigeria. Eur J Gastroenterol Hepatol.

[9] Thompson WG (2002). Irritable bowel syndrome and coeliac disease. Lancet.

[10] Khademolhosseini F, Mehrabani D, Nejabat M, Beheshti M, Heydari ST, Mirahmadizadeh A (2011). Irritable bowel syndrome in adults over 35 years in Shiraz, southern Iran: prevalence and associated factors. J Res Med Sci.

[11] Thompson WG, Heaton KW, Smyth GT, Smyth C (2000). Irritable bowel syndrome in general actice: prevalence, characteristics, and referral. Gut.

[12] Khoshkrood-Mansoori B, Pourhoseingholi MA, Safaee A, Moghimi-Dehkordi B, Sedigh-Tonekaboni B, Pourhoseingholi A (2009). Irritable bowel syndrome: a population based study. J Gastrointestin Liver Dis.

[13] Eriksson EM, Andren KI, Eriksson HT, Kurlberg GK (2008). Irritable bowel syndrome subtypes differ in body awareness, psychological symptoms and biochemical stress markers. World J Gastroenterol.

[14] Drossman DA, Creed FH, Olden KW, Svedlund J, Toner BB, Whitehead WE (1999). Psychosocial aspects of the functional gastrointestinal disorders. Gut.

[15] Walker EA, Katon WJ, Jemelka RP, Roy-Bryne PP (1992). Comorbidity of gastrointestinal complaints, depression, and anxiety in the Epidemiologic Catchment Area (ECA) Study. Am J Med.

[16] Fock KM, Chew CN, Tay LK, Peh LH, Chan S, Pang EP (2001). Psychiatric illness, personality traits and the irritable bowel syndrome. Ann Acad Med Singapore.

[17] Porcelli P, Todarello O (2007). Psychological factors affecting functional gastrointestinal disorders. Adv Psychosom Med.

[18] Talley NJ, Dennis EH, Schettler-Duncan VA, Lacy BE, Olden KW, Crowell MD (2003). Overlapping upper and lower gastrointestinal symptoms in irritable bowel syndrome patients with constipation or diarrhea. Am J Gastroenterol.

[19] Balboa A, Mearin F, Badia X, Benavent J, Caballero AM, Dominguez-Munoz JE (2006). Impact of upper digestive symptoms in patients with irritable bowel syndrome. Eur J Gastroenterol Hepatol.

[20] Gwee KA, Leong YL, Graham C, McKendrick MW, Collins SM, Walters SJ (1999). The role of psychological and biological factors in postinfective gut dysfunction. Gut.

[21] Sorouri M, Pourhoseingholi MA, Vahedi M, Safaee A, Moghimi-Dehkordi B, Pourhoseingholi A Functional bowel disorders in Iranian population using Rome III criteria. Saudi J Gastroenterol.

[22] Solhpour A, Safaee A, Pourhoseingholi MA, Moghimi-Dehkordi B, Habibi M, Qafarnejad F Relationship between uninvestigated dyspepsia and body mass index: a population-based study. East Afr J Public Health.

[23] Barzkar M, Pourhoseingholi MA, Habibi M, Moghimi-Dehkordi B, Safaee A, Pourhoseingholi A ( 2009). Uninvestigated dyspepsia and its related factors in an Iranian community. Saudi Med J.

[24] Moghimi-Dehkordi B, Safaee A, Vahedi M, Pourhoseingholi MA, Habibi M, Pourhoseingholi A (2011). Association between perceived depression, anxiety and stress with Body Mass Index: results from a community-based cross-sectional survey in Iran. Italian J Public Health.

[25] Ross R, Zeller R, Srisaeng P, Yimmee S, Somchid S, Sawatphanit W (2005). Depression, stress, emotional support, and self-esteem among baccalaureate nursing students in Thailand. Int J Nurs Educ Scholarsh.

[26] Brommelhoff JA, Conway K, Merikangas K, Levy BR (2004). Higher rates of depression in women: role of gender bias within the family. J Womens Health (Larchmt).

[27] Ferguson A, Sircus W, Eastwood MA (1977). Frequency of "functional" gastrointestinal disorders. Lancet.

[28] Thompson WG (1999). Irritable bowel syndrome: a management strategy. Baillieres Best Pract Res Clin Gastroenterol.

[29] Ford MJ, Miller PM, Eastwood J, Eastwood MA (1987). Life events, psychiatric illness and the irritable bowel syndrome. Gut.

[30] Blewett A, Allison M, Calcraft B, Moore R, Jenkins P, Sullivan G (1996). Psychiatric disorder and outcome in irritable bowel syndrome. Psychosomatics.

[31] Drossman DA, McKee DC, Sandler RS, Mitchell CM, Cramer EM (1998). The relationship between irritable bowel syndrome and psychiatric illness. A family study. Psychosomatics.

[32] Drossman DA, McKee DC, Sandler RS, Mitchell CM, Cramer EM, Lowman BC (1988). Psychosocial factors in the irritable bowel syndrome. A multivariate study of patients and nonpatients with irritable bowel syndrome. Gastroenterology.

[33] Lydiard RB, Fossey MD, Marsh W, Ballenger JC (1993). Prevalence of psychiatric disorders in patients with irritable bowel syndrome. Psychosomatics.

[34] Tollefson GD, Luxenberg M, Valentine R, Dunsmore G, Tollefson SL (1991). An open label trial of alprazolam in comorbid irritable bowel syndrome and generalized anxiety disorder. J Clin Psychiatry.

[35] Masand PS, Kaplan DS, Gupta S, Bhandary AN, Nasra GS, Kline MD (1995). Major depression and irritable bowel syndrome: is there a relationship?. J Clin Psychiatry.

[36] Gupta S, Masand PS, Kaplan D, Bhandary A, Hendricks S (1997). The relationship between schizophrenia and irritable bowel syndrome (IBS). Schizophr Res.

[37] Lydiard RB, Laraia MT, Howell EF, Ballenger JC (1986). Can panic disorder present as irritable bowel syndrome?. J Clin Psychiatry.

[38] Whitehead WE, Crowell MD (1991). Psychologic considerations in the irritable bowel syndrome. Gastroenterol Clin North Am.

[39] Miller AR, North CS, Clouse RE, Wetzel RD, Spitznagel EL, Alpers DH (2001). The association of irritable bowel syndrome and somatization disorder. Ann Clin Psychiatry.

[40] O'Sullivan MA, Mahmud N, Kelleher DP, Lovett E, O'Morain CA (2000). Patient knowledge and educational needs in irritable bowel syndrome. Eur J Gastroenterol Hepatol.

[41] Cann PA, Read NW, Brown C, Hobson N, Holdsworth CD (1983). Irritable bowel syndrome: relationship of disorders in the transit of a single solid meal to symptom patterns. Gut.

[42] Grundy D, Al-Chaer ED, Aziz Q, Collins SM, Ke M, Taché Y (2006). Fundamentals of neurogastroenterology: basic science. Gastroenterology.

[43] Wood JD, Alpers DH, Andrews PL (1999). Fundamentals of neurogastroenterology. Gut.

[44] Aggarwal A, Cutts TF, Abell TL, Cardoso S, Familoni B, Bremer J (1994). Predominant symptoms in irritable bowel syndrome correlate with specific autonomic nervous system abnormalities. Gastroenterology.

